# Disinfection of Instruments

**Published:** 1895-02

**Authors:** 


DISINFECTION OF INSTRUMENTS.—Sterilize coarse building sand by roasting. Fill a suitable vessel with this, and pour in a sublimate solution 4 per cent, or lysol 50 per cent, till the sand is thoroughly soaked. Keep covered with a sterilized piece of pasteboard. Pass all instruments through this two or three times. Offers simplicity, rapidity, absolute sterility, no injury to instruments.—*Items of Interest.*

